# Obesity, Cardiovascular Fitness, and Inhibition Function: An Electrophysiological Study

**DOI:** 10.3389/fpsyg.2016.01124

**Published:** 2016-07-27

**Authors:** Tai-Fen Song, Lin Chi, Chien-Heng Chu, Feng-Tzu Chen, Chenglin Zhou, Yu-Kai Chang

**Affiliations:** ^1^Graduate Institute of Athletics and Coaching Science, National Taiwan Sport UniversityTaoyuan, Taiwan; ^2^Department of Exercise and Health Promotion, Ta Hwa University of TechnologyHsin-chu, Taiwan; ^3^School of Kinesiology, Shanghai University of SportShanghai, China

**Keywords:** obese, fitness, Stroop test, P3, N1

## Abstract

The purpose of the present study was to examine how obesity and cardiovascular fitness are associated with the inhibition aspect of executive function from behavioral and electrophysiological perspectives. One hundred college students, aged 18–25 years, were categorized into four groups of equal size on the basis of body mass index and cardiovascular fitness: a normal-weight and high-fitness (NH) group, an obese-weight and high-fitness (OH) group, a normal-weight and low-fitness (NL) group, and an obese-weight and low-fitness (OL) group. Behavioral measures of response time and number of errors, as well as event-related potential measures of P3 and N1, were assessed during the Stroop Task. The results revealed that, in general, the NH group exhibited shorter response times and larger P3 amplitudes relative to the NL and OL groups, wherein the OL group exhibited the longest response time in the incongruent condition. No group differences in N1 indices were also revealed. These findings suggest that the status of being both normal weight and having high cardiovascular fitness is associated with better behavioral and later stages of electrophysiological indices of cognitive function.

## Introduction

Obesity, an emerging global pandemic, has not only been found to be associated with various health problems, including cardiovascular disease, diabetes, stroke, and high blood pressure (Nilsson and Nilsson, 2009; Ng et al., 2014), but has also been found to be associated with psychological and social issues such as a distorted body image and poorer interpersonal relationships (Edmunds et al., 2001). Moreover, harmful obesity-related outcomes have also been reported to extend to cognitive function. Specifically, obesity has been found to be associated with an elevated risk of impaired cognitive function (Gunstad et al., 2010; Hsu et al., 2015), and this negative relationship between obesity and cognitive function has been demonstrated in people of various ages, including children, adolescents (Liang et al., 2014; Martin et al., 2014), and even older adults (Siervo et al., 2011).

Executive function, one aspect of cognitive profiles that has been found to be linked to obesity, has received a substantial amount of research attention (Fitzpatrick et al., 2013; Reinert et al., 2013). Executive function is believed to reflect higher-order and effortful cognitive control that serves to manage and supervise multiple basic cognitive processes in order to achieve purposeful or goal-directed behaviors (Alvarez and Emory, 2006; Etnier and Chang, 2009), with the frontal lobe believed to be heavily involved (Alvarez and Emory, 2006; Anderson et al., 2008). Interestingly, obese status has been observed to be associated with neurostructural deficits in the prefrontal and orbitofrontal cortices (Reinert et al., 2013) and in the frontal-subcortical activation of cognitive function (Stanek et al., 2013), suggesting a physiological linkage between obesity and executive dysfunction.

Rather than being a singular concept, executive function is thought to involve multiple subdomains, including inhibition, updating, and shifting (Miyake et al., 2000), with the inhibition aspect of executive function having been examined most often in obesity-related studies (Reinert et al., 2013). Inhibition refers to the ability to focus limited attention on relevant information or to resolve conflicting responses from unrelated or distracting stimuli (Heinemann et al., 2009; Wostmann et al., 2013). Kamijo et al. (2012a) indicated that higher body weight and fat mass, as assessed, respectively, by body mass index (BMI) and dual-energy X-ray absorptiometry, are negatively associated with performance on cognitive tasks requiring substantial inhibitory control, but not tasks requiring only limited degrees of inhibition, a finding which suggests that obesity is particularly relevant to inhibition. Similar findings indicating an association between obesity and impaired inhibition have also been reported (Kamijo et al., 2012b; Nederkoorn et al., 2012; Wirt et al., 2014).

One factor that has been found to have a protective effect on executive function is cardiovascular fitness; the positive association between the fitness and cognitive function has been reported by several meta-analytic reviews (Sibley and Etnier, 2003; Angevaren et al., 2008; Smith et al., 2010; Scherder et al., 2014). Additionally, high levels of cardiovascular fitness have been observed to have benefits with regard to multiple cognitive functions, with larger effects having been observed on executive function (Colcombe and Kramer, 2003). Research has also shown a robust relationship between cardiovascular fitness and inhibition. For example, Dupuy et al. (2015) found that highly fit females, regardless of whether they are young or old, exhibit better inhibition performance on the Stroop Task than those with low-fitness status. Other studies employing longitudinal designs also demonstrated better Stroop Task performances following an aerobic exercise training program lasting either 10 months (Smiley-Oyen et al., 2008) or 16 weeks (Vaughan et al., 2014) compared to non-exercise control interventions. While a positive effect of cardiovascular fitness on inhibition has thus been suggested, it should be noted that these studies generally examined normal-weight, older populations. As such, whether cardiovascular fitness can moderate the relationship between obesity and inhibition remains unknown.

The relationship between fitness and executive function has been investigated through the measurement of event-related potentials (ERPs). More specifically, the term “ERP” refers both to the electrical activity of the brain connected to a specific task event or response and the neuroelectrical or electrophysiological technique that offers precise temporal resolution of such neural processes underlying task performance (Fabiani et al., 2009). ERP studies investigating fitness and executive function have generally reported a relationship between higher levels of cardiovascular fitness and increased P3 amplitude during the performance of executive function tasks (Hillman et al., 2004, 2006; Pontifex et al., 2011; Chang et al., 2013). This association between P3 amplitudes and fitness may be of particular importance to the obese population. Tascilar et al. (2011) examined cognitive function and related P3 alterations among children with and without obesity and found that obese children demonstrated decreased P3 amplitudes compared to their non-obese counterparts. When they further categorized those obese children into sub-groups consisting of those with or without insulin resistance, greater decreases in P3 amplitudes were observed in the obese children with insulin resistance than among the children without such resistance, suggesting that obesity is associated with impaired electrophysiological activity.

Studies of the relationship between fitness and ERPs have typically placed an emphasis on the P3 component. However, studies of other, less-examined ERP components, such as the N1 component, have reported mixed results. For example, Chang et al. (2013) observed that highly active older adults demonstrated not only larger P3 amplitudes but also larger N1 amplitudes during an update task relative to those with low activity levels. However, in a study of younger adults with high or low fitness levels, only P3 (but not N1) amplitudes during a shifting task were found to be significantly different between the two groups (Scisco et al., 2008). These inconsistent findings may be attributable to the heterogeneous methods employed by the different studies, so further investigations of fitness and its relationship, if any, to the N1 are required. Relatedly, to our knowledge, no previous study has examined the relationships between fitness, inhibition, and N1 in both normal- and obese-weight populations.

Taken together, obesity and cardiovascular fitness have been associated, respectively, with weakened and strengthened inhibitory capacity in executive function. However, few studies have explored the relationships among cardiovascular fitness, obesity, and inhibition simultaneously. Furthermore, how cardiovascular fitness and obesity are associated with multiple electrophysiological activities during inhibition is not yet well-understood. The purpose of the present study, then, was to examine the relationship between cardiovascular fitness and inhibition in both obese and non-obese adults, with both behavioral and electrophysiological activity in terms of the P3 and N1 components of ERPs being investigated. It was expected that obese individuals would exhibit worse inhibition-related performances in terms of both behavioral and electrophysiological measures than the normal-weight individuals. It was also expected that the relationship between obesity and inhibition would be moderated by cardiovascular fitness in those obese individuals who had high cardiovascular fitness.

## Materials and Methods

### Participants

Male university students between 18 and 25 years of age were recruited via internet advertisements and flyers posted in communities and health centers in cities in northern Taiwan. These potential participants were required to meet the following initial inclusion criteria: (a) normal or corrected-to-normal vision, (b) no color blindness, (c) no history of neurological disorders or cardiovascular disease, and (d) the capacity to complete a cardiovascular fitness assessment measured by the Physical Activity Readiness Questionnaire (PAR-Q). Potential participants meeting those requirements were also required to meet the additional criterion of being either normal-weight (BMI = 18.5–24 kg/m^2^) or obese (BMI > 27 kg/m^2^). The determination of an individual participant’s weight status was based on the BMI norms for adults published by the Ministry of Health and Welfare, Taiwan. Finally, potential participants also had to meet the criterion of having either a high (maximal oxygen uptake measures, VO_2max_ > 65th percentile) or low (VO_2max_ < 35th) fitness level. The cardiovascular fitness status of the participants was determined according to normative data provided by the American College of Sports Medicine (2013). One hundred participants met the above inclusion criteria and were then placed into one of the following four groups: the normal-weight and high-fitness (NH) group, the obese-weight and high-fitness (OH) group, the normal-weight and low-fitness (NL) group, or the obese-weight and low-fitness (OL) group. **Table 1** summarizes the participants’ demographic information. All the participants completed a written informed consent form in accordance with the requirements of the Institutional Review Board of National Taiwan University.

**Table 1:** Demographic and cardiovascular fitness characteristics among four groups (mean ± SD).

Variables	NH (*n* = 25)	OH (*n* = 25)	NL (*n* = 25)	OL (*n* = 25)
Age (years)	21.00 ± 1.80	20.84 ± 2.30	21.96 ± 2.19	21.16 ± 2.23
Height (cm)	174.00 ± 4.06	178.36 ± 8.43	175.84 ± 6.66	173.88 ± 5.99
Digit Span test				
Forward	14.88 ± 0.83	14.24 ± 1.17	14.52 ± 1.26	14.12 ± 1.17
Backward	10.00 ± 3.11	7.84 ± 2.78	8.44 ± 2.79	8.36 ± 3.09
Weight				
Weight (kg)	64.64 ± 5.28^a^	95.44 ± 17.18^b^	66.92 ± 7.42^a^	101.20 ± 17.14^b^
BMI (kg/m^2^)	21.34 ± 1.42^a^	29.80 ± 3.32^b^	21.59 ± 1.44^a^	33.42 ± 5.23^b^
Waist/hip ratio	0.78 ± 0.04^a^	0.87 ± 0.05^b^	0.82 ± 0.04^a^	0.91 ± 0.04^b^
Body fat mass (%)	13.97 ± 3.70^a^	24.28 ± 3.87^b^	16.01 ± 3.80^a^	30.10 ± 4.01^b^
VO_2max_ (ml/kg/min)	54.66 ± 4.81^a^	51.96 ± 3.44^a^	35.77 ± 2.89^b^	36.23 ± 3.49^b^

*NH, normal-weight and high-fit; OH, obese-weight and high-fit; NL, normal-weight and low-fit; OL, obese-weight and low-fit; VO_2max_, estimatedVO_2max._^a,b^Significant differences between groups, p < 0.05.*

### Cardiovascular Fitness Assessment

The cardiovascular fitness (indexed as estimated VO_2max_) of each participant was estimated using a YMCA cycling ergometer submaximal exercise test (Golding et al., 1989). The protocol was designed for adults with a Class A risk stratification (Fletcher et al., 2001) and consists of a series of 2–4 consecutive 3-min cycling stages. Through the whole testing process, the participant’s heart rate (HR) was continuously monitored by a Polar heart rate monitor (Sport Tester PE 3000, Polar Electro Oy, Kempele, Finland). Participants were instructed to sit and pedal at a constant speed of 50 rpm with a workload of 150 kpm/min (25 W) on an electronically braked cycle ergometer (Ergoselect 100/200 Ergoline GmbH, Germany) for the first 3-min stage. After the initial 3-min stage, the workload was increased in accordance with the given participant’s steady-state HR recorded during the last 15–30 s of the first 3-min stage. For instance, if the participant’s HR was less than 80 bpm, then the workloads for the second and third 3-min stages would be set to 125 W (750 kpm/min) and 150 W (900 kpm/min), respectively. An additional 3-min cycling stage (at 150 kpm/min or 25 W) would be added if the participant’s target HR, which was 85% of the participant’s age-predicted HRmax, was not achieved. A numerical rating of perceived exertion (RPE) from the Borg 6 to 20 (Borg, 1982) scale was provided by the participant every 2 min during the cardiovascular fitness assessment.

### Stroop Task

The study also employed a computer-based modified Stroop Color-Word test (Stroop, 1935), a widely utilized neuropsychological test of both basic information processing and executive function. Two Stroop conditions, the incongruent and neutral conditions, were employed. For the incongruent condition, the visual stimuli were Chinese words (e.g., 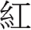 [Red], 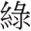 [Green], and 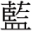 [Blue]). However, the semantic meaning of the given word and the actual pixel color of it as it appeared on the screen were inconsistent in the incongruent condition (e.g., the word 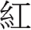 [Red] was shown in blue color). For the neutral condition, colored squares of red, green, or blue were used as the stimuli. A single test block consisted of 36 incongruent and 36 neutral stimuli with a mixed and random order presentation. Participants were required to complete six blocks (432 trials in total), with a 2-min rest between each block. Each individual trial began with a fixation cross being displayed for 600 ms, followed by the presentation of the given 2-cm stimulus for 500 ms in the center of a 17-inch computer screen. Participants were instructed to respond in accordance with the color of the stimulus as quickly and accurately as possible by pressing one of the three color response buttons on a response box (10 cm × 8 cm × 2 cm box). Each trial was completed once a response was made between 200 and 1000 ms after the display of the stimulus or if no response was made within 1000 ms. The response time and number of errors of each correct response were futher identified and recorded as behavioral indices.

### Electrophysiological Recording and Analysis

Continuous scalp electroencephalographic (EEG) activity measurements were collected using the Neuroscan SynAmps^2^ system Scan (Scan 4.0, Compumedics Neuroscan) via an elastic cap with 32 Ag/AgCl electrodes arranged according to the international standard 10–10 system (Quick-cap, Neuroscan, Inc., Lexington, VA, USA). A suitable elastic cap was chosen to fit each individual participant’s head size and each electrode was filled with electro-gel to reduce the impedance of each electrode to 5 kΩ. The recording was re-referenced to the averaged mastoids, with AFz serving as the ground electrode. Adhesive electrodes were placed below and above the left orbit and on the outer canthus of each eye to monitor bipolar electrooculography (EOG) activity. With a low cutoff value (70 Hz) and high cutoff value (0.05 Hz), the data were digitized at an A/D rate of 500 Hz with a 60 Hz notch filter.

Oﬄine EEG data were initially processed to remove the ocular artifact and segmented into epochs for time-locked ERP components. The epoch was set to last from 200 ms before and 1000 ms after the onset of the stimulus. The baseline correction was conducted using the 200 ms pre-stimulus period. Data were then filtered using a low-pass shift 30 Hz (12 dB/octave). Any trials with a response error or artifact exceeding ±100 μV were rejected before averaging. The remaining effective data were then averaged. The P3 and N1 components from each correct response were identified and recording as electrophysiological indices. The time windows for peak detection of the P3 and N1 components were 300–700 ms (positive-going peak) and 100–150 ms (negative-going peak), respectively. The ERP amplitude and latency from Fz, Cz, and Pz were collected for further statistical analysis (Hillman et al., 2005; Themanson and Hillman, 2006; Chang et al., 2013; Dai et al., 2013). Scalp topographies based upon 32 electrodes for N1 and P3 components across the four groups were also provided.

### Experimental procedure

Each participant was required to visit the laboratory located on the National Taiwan Sport University campus individually on two occasions within a 7-days interval. On the first visit, a brief introduction regarding the experimental procedure was provided and the participant then filled out the informed consent form. Next, the participant’s demographic information, BMI, and working memory aspect of the intelligence quotient assessed by the Digit Span test of the Wechsler Adult Intelligence Scale-Third Edition (WAIS-III; Wechsler, 1997) were acquired. The participant was then fitted with a Polar heart rate monitor and instructed to perform the YMCA cycling ergometer submaximal exercise test. The participants were only required to visit the laboratory twice if they met the cardiovascular fitness and obesity status criteria.

On their second visit, the qualified participants were instructed to be comfortably seated in a chair in a dimly lit, sound-proof, electrically shielded room. Prior to the official test, each participant was instructed to practice the Stroop Task until a correct response rate of 85% was achieved, during which time the electrophysiological recording settings were prepared. The official Stroop Task was then administered and EEG recordings were taken throughout the task performance period. Each participant was compensated with a payment of approximately US $30 dollars for participating in the experiment.

### Statistical Analysis

Statistical analyses were performed using SPSS version 21.0 (IBM, Corp., Armonk, NY, USA). Demographic data were analyzed using the one-way analysis of variance (ANOVA) to examine any potential demographic differences among the four groups. For behavioral data, a 4 (Group: NH, OH, NL, OL) × 2 (Stroop condition: neutral, incongruent) mixed-model ANOVA was employed for response time and number of errors, respectively. For the ERP components, a 4 (Group) × 2 (Stroop condition) × 3 (Site: Fz, Cz, Pz) mixed-model ANOVA was employed to analyze P3 amplitude, P3 latency, N1 amplitude, and N1 latency, respectively. The Greenhouse-Geisser method that was utilized to correct for violations of the sphericity assumption. *Post hoc* Student-Newman–Keuls and multiple *t*-test comparisons with Bonferroni correction were conducted where appropriate. The partial eta squared (η^2^) was reported for significant effects, as determined by *p* < 0.05, before any statistical adjustment.

## Results

### Participant Characteristics

One-way ANOVAs revealed no significant differences among the four groups in age, height, or Digit Span test scores. However, the analysis revealed significant differences among the four groups in weight [*F*(3,96) = 53.50, *p* < 0.001], BMI [*F*(3,96) = 86.00, *p* < 0.001], waist/hip ratio [*F*(3,96) = 43.02, *p* < 0.001], body fat mass [*F*(3,96) = 94.59, *p* < 0.001] and VO_2max_ [*F*(3,96) = 182.24, *p* < 0.001]. *Post hoc* Student-Newman–Keuls tests revealed that the OH and OL groups had higher values of weight, BMI, waist/hip ratio, and body fat mass than the NH and NL groups (*ps* < 0.05), while the NH and OH groups had higher estimated VO_2max_ values than the NL and OL groups (*ps* < 0.05; **Table 1**).

### Behavioral Data

#### Response Time

A two-way ANOVA revealed a main effect of the group [*F*(3,96) = 9.25, *p* < 0.001, $\eta_{\text{p}}^{2}=0.22$]. A *post hoc* Newman–Keuls test revealed that shortest response time was observed for the NH group (474.46 ± 16.76 ms), followed by the OH group (526.53 ± 16.76 ms) and the NL group (542.79 ± 16.76 ms), while the longest response time was observed for the OL group (598.25 ± 16.76 ms, *p*s < 0.05).

The analysis also revealed a main effect of the Stroop condition [*F*(1,96) = 250.69, *p* < 0.001, $\eta_{\text{p}}^{2}=0.72$]. The follow-up comparison revealed that a shorter response time was observed for the neutral (492.57 ± 7.53 ms) condition as compared to the incongruent condition (578.45 ± 9.92 ms, *p* < 0.001).

**FIGURE 1:**
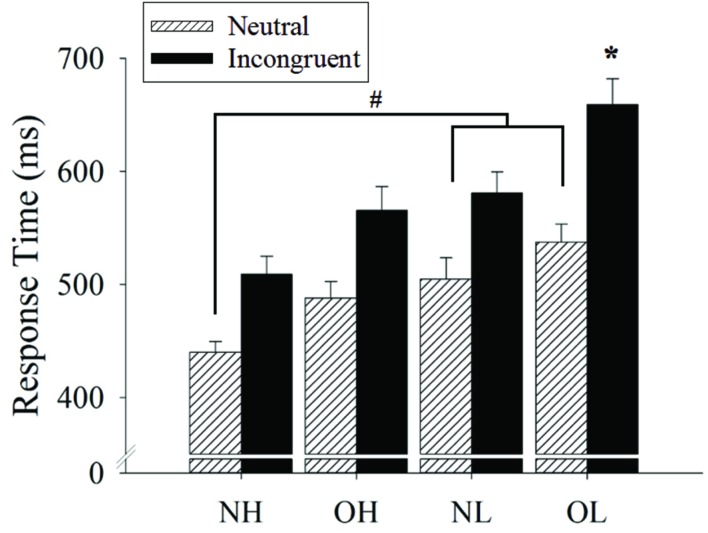
**Response time (mean ± SE) of Stroop neutral and incongruent conditions across four groups: normal-weight and high-fit (NH), obese-weight and high-fit (OH), normal-weight and low-fit (NL), and obese-weight and low-fit (OL)**. Note that there is a main effect of the Stroop condition, with a shorter response time for the neutral condition as compared to the incongruent condition. ^#^Represents the significant difference in neutral condition. ^∗^Represents the significant difference in incongruent condition.

An interaction of group and Stroop condition was also observed [*F*(3,96) = 4.83, *p* < 0.01, $\eta_{\text{p}}^{2}=0.13$]. The follow-up comparison revealed a shorter response time for the NH group (440.15 ± 15.06 ms) relative to NL and OL groups (504.70 ± 15.06 and 537.68 ± 15.06 ms, *ps* < 0.05) for the neutral condition. For the incongruent condition, the OL group had the longest response time (658.81 ± 19.85 ms), followed by the NL, OH, and NH groups (580.89 ± 19.85, 565.29 ± 19.85, and 508.78 ± 19.85 ms, *p*s < 0.05), but no significant differences were observed among the latter three groups. In addition, all four groups had a shorter response time for the neutral as compared to the incongruent condition (*ps* < 0.001; **Figure 1**).

#### Number of Errors

The analysis revealed no main effect of group, but did reveal a main effect of Stroop condition [*F*(1,96) = 58.72, *p* < 0.001, $\eta_{\text{p}}^{2}=0.38$]. The follow-up comparison revealed that there were fewer errors for the neutral condition (91.40 ± 0.01%) than for the incongruent condition (86.10 ± 0.01%). No significant interaction of group and Stroop condition was observed (*F* = 1.93, *p* > 0.05).

### ERP Data

**FIGURE 2:**
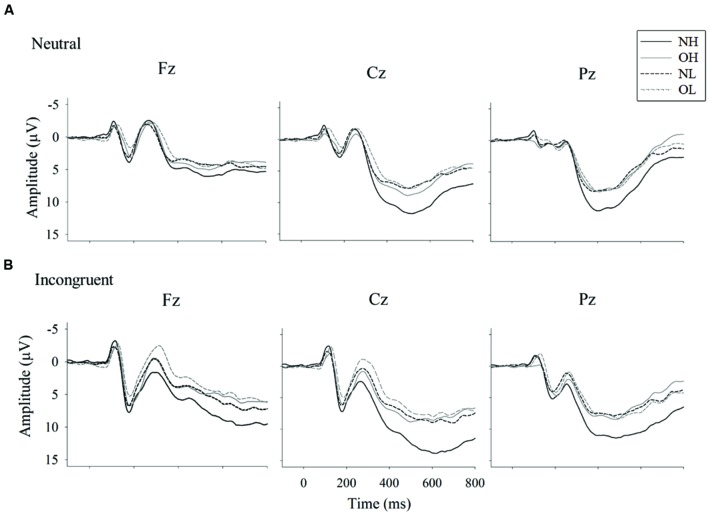
**Grand-averaged ERP waveform for Stroop neutral **(A)** and incongruent **(B)** conditions **(B)** across four groups: normal-weight and high-fit (NH), obese-weight and high-fit (OH), normal-weight and low-fit (NL), and obese-weight and low-fit (OL)**.

**FIGURE 3:**
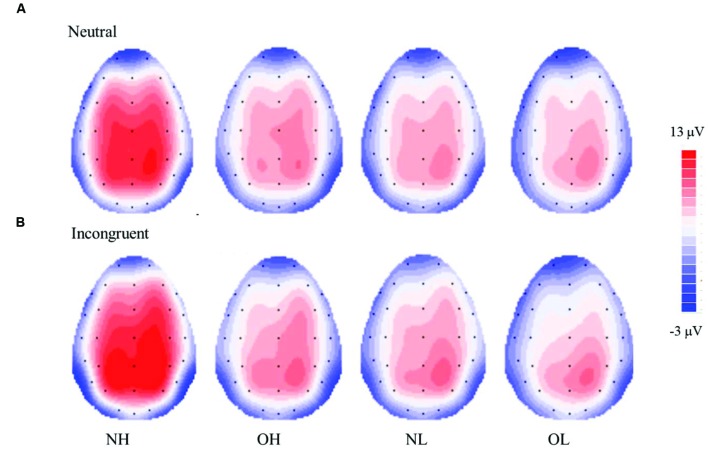
**Topographic distribution of the mean P3 voltage (300–550 ms) for the Stroop neutral **(A)** and incongruent **(B)** conditions across four groups; normal-weight and high-fit (NH), obese-weight and high-fit (OH), normal-weight and low-fit (NL), and obese-weight and low-fit (OL)**.

**Figure 2** illustrates the grand-averaged ERP waveforms and **Figure 3** illustrates the topographic distributions of the P3 voltages across the four groups.

#### N1 Amplitude

The three-way ANOVA revealed no main effect of group, but it did reveal main effects of Stroop condition [*F*(1,96) = 53.26, *p* < 0.001, $\eta_{\text{p}}^{2}=0.36$] and site [*F*(2,192) = 30.09, *p* < 0.001, $\eta_{\text{p}}^{2}=0.24$]. The follow-up comparison revealed that a larger N1 amplitude was observed for the incongruent condition (-2.56 ± 0.29 μV) than for the neutral condition (-1.55 ± 0.30 μV, *p* < 0.001). In addition, larger N1 amplitudes were observed for the Fz (-2.43 ± 0.31 μV) and Cz (-2.31 ± 0.31 μV) sites as compared to the Pz site (-1.43 ± 0.27 μV). No other significant effects were observed.

#### N1 Latency

The analysis revealed no main effect of group, Stroop condition, or site, in addition to revealing no two- or three-way interaction effects (*ps* > 0.05).

#### P3 Amplitude

The analysis revealed a main effect of the group [*F*(3,96) = 6.67, *p* < 0.001, $\eta_{\text{p}}^{2}=0.17$]. *Post hoc* Student-Newman–Keuls tests revealed a larger P3 amplitude for the NH group (12.69 ± 0.82 μV) than for the NL (9.06 ± 0.82 μV), OH (8.60 ± 0.82 μV), and OL groups (8.02 ± 0.82 μV, *p* < 0.05).

The analysis also revealed significant main effects of Stroop condition [*F*(1,96) = 22.80, *p* < 0.001, $\eta_{\text{p}}^{2}=0.19$] and site [*F*(2,192) = 63.64, *p* < 0.001, $\eta_{\text{p}}^{2}=0.40$]. The follow-up comparison revealed that a larger P3 amplitude was observed for the incongruent condition (10.08 ± 0.44 μV) than for the neutral condition (9.11 ± 0.40 μV, *ps* < 0.001). In addition, the largest P3 amplitude was observed for the Cz site (10.99 ± 0.49 μV), followed by the Pz site (10.13 ± 0.41 μV), and the Fz site (7.65 ± 0.43 μV, *p*s < 0.01).

An interaction of group and Stroop condition was observed [*F*(3,96) = 2.72, *p* < 0.05, $\eta_{\text{p}}^{2}=0.08$]. The follow-up comparison showed that the NH group had a larger P3 amplitude than the NL, OH, and OL groups in both the neutral (11.78 ± 0.80 vs. 8.46 ± 0.80, 8.35 ± 0.80 and 7.84 ± 0.80 μV, *ps* < 0.05) and incongruent conditions (13.60 ± 0.88 vs. 9.66 ± 0.88, 8.86 ± 0.88 and 8.20 ± 0.88 μV, *ps* < 0.05), with no significant differences among the latter three groups. Both the NH and NL groups had a larger P3 amplitude in the incongruent condition than in the neutral condition (*p* < 0.01), whereas the same difference was not observed in the OH and OL groups.

An interaction between Stroop condition and site was also observed [*F*(2,192) = 10.93, *p* < 0.001, $\eta_{\text{p}}^{2}=0.10$]. The follow-up comparisons revealed that the incongruent condition had larger P3 amplitudes than the neutral condition at the Fz (8.35 ± 0.48 vs. 6.96 ± 0.41 μV, *p* < 0.001), Cz (11.49 ± 0.53 vs. 10.49 ± 0.48 μV, *p* < 0.001), and Pz sites (10.39 ± 0.44 vs. 9.87 ± 0.41 μV, *p* < 0.05). The Fz site had a smaller P3 amplitude than the Cz and Pz sites (*ps* < 0.001) for the incongruent condition, whereas the Fz site had the smallest P3 amplitude, followed by the Pz and Cz sites, for the neutral condition (*ps* < 0.01).

#### P3 Latency

The analysis revealed only significant main effects of Stroop condition [*F*(1,96) = 78.82, *p* < 0.001, $\eta_{\text{p}}^{2}=0.45$] and site [*F*(2,192) = 96.59, *p* < 0.001, $\eta_{\text{p}}^{2}=0.50$]. The follow-up comparison revealed a longer P3 latency for the incongruent condition (546.19 ± 10.64 ms) than for the neutral condition (477.94 ± 9.64 ms, *p* < 0.001). In addition, the longest P3 latency was observed for the Fz site (552.72 ± 11.17 ms), followed by the Cz site (528.13 ± 10.29 ms), and the Pz site (455.36 ± 9.35; *ps* < 0.001).

## Discussion

The current study is among the first to examine how obesity and cardiovascular fitness affect the inhibition aspect of executive function from behavioral and electrophysiological perspectives. Our primary behavioral findings generally revealed that the NH and OL group demonstrated the shortest and longest response time for the Stroop Task, respectively. In addition, the OL group demonstrated the longer response time relative to the NH in the neutral condition and longest response time compared to other three groups (i.e., NL, OH, and NH) in the incongruent condition. In terms of ERP measurements, the NH group demonstrated a larger Stroop Task-induced P3 amplitude than the other three groups, while there were no significant differences among the other three groups. However, no group differences were observed for the N1 indices across the four groups.

### Obesity, Cardiovascular Fitness, and Behavioral Measures

Our results showing longer response times and more errors for the incongruent condition than for the neutral condition indicated a typical phenomenon known as the “Stroop Effect” (Chang et al., 2015a,b). That is, the incongruent Stroop condition required more time from the involvement of executive function to inhibit or over-ride prepotent responses than did the neutral condition, a finding that reflects basic information processes (Friedman et al., 2009). The presentation of the Stroop Effect also suggested that our modified Stroop Task was appropriately manipulated.

Shorter response times in the neutral condition for high cardiovascular fitness relative to low fitness were observed regardless of whether the individuals were normal-weight (i.e., the NH and NL groups) or obese (i.e., the OH and OL groups). Although, OL and NL demonstrated longer response time compared to NH, no differences were observed in individuals in high cardiovascular fitness (i.e., the NH and OH groups) or low cardiovascular fitness (i.e., the NL and OL groups). These results suggested that while obesity may be associated with slower basic information processing and fitness has larger influence in the processes. Fitness is related to increase the processing speed replicated those of previous obesity studies that examined other cognitive demands such as complex attention, verbal memory, and visual memory (Prickett et al., 2014), as well as a fitness study that examined a normal-weight population (Buck et al., 2008). At the same time, the results of the present study extend the knowledge base regarding associations between obesity, cardiovascular fitness, and Stroop task-related basic information processing.

In terms of the incongruent condition, OL group had worse performance compared to other three groups (i.e., the OH, NL, and OL groups), with no significant difference being observed among the groups. This fact suggests that individual with both more obese and less fitness has a crucial influence on inhibition. These results are partly consistent with those of previous studies that reported a negative relationship between obesity and executive function (Fitzpatrick et al., 2013; Reinert et al., 2013; Miller et al., 2015). A notable study that utilized both cross-sectional and longitudinal perspectives previously demonstrated the positive association between cardiovascular fitness and executive function (Colcombe and Kramer, 2003; Chaddock et al., 2011). The beneficial effects of higher cardiovascular fitness have been linked to the greater brain volumes associated with better executive function (Erickson et al., 2009; Weinstein et al., 2012; Voss et al., 2013) and to the proliferation of new neurons or related molecules (e.g., *N*-acetylaspartate levels) associated with cognitive function (Erickson et al., 2012). Notably, our exploration extended the current understanding regarding how both obesity and cardiovascular fitness affects executive function.

### Obesity, Cardiovascular Fitness, and Electrophysiological Measures

The ERP measures were partly consistent with the behavioral performances; that is, the group with normal weight and high cardiovascular fitness showed the largest P3 amplitude compared to the other three groups. However, this largest P3 amplitude was found in the NH group for both the neutral and incongruent conditions of the Stroop Task. Our finding of a larger P3 amplitude in individuals with higher fitness compared to those with lower fitness was in line with other studies of ERP associated with fitness. For example, previous studies have found that individuals with higher fitness or who regularly engage in exercise demonstrated a larger P3 amplitude during a visual oddball task (Hillman et al., 2005) and an executive function-related task (Pontifex et al., 2011; Dai et al., 2013; Fong et al., 2014) than their less fit or less active counterparts. P3 amplitude is believed to reflect proportionally the amount of resources allocated toward the suppression of extraneous neuronal activity in order to facilitate attention (Polich, 2007). As such, it is plausible that the fitness level is associated with greater engaged attention to multiple aspects of cognitive domains.

While individuals with normal weight and high cardiovascular fitness showed a larger P3 amplitude compared to the obese individuals, it seems that cardiovascular fitness itself had a limited effect on the obese individuals, given that no P3 amplitude difference was observed between the OH and OL groups. These findings also suggest that obesity itself has a prominent effect in terms of impairing cognitive function as indicated by electrophysiological measurements. Given the previous evidence indicating that obesity is associated with lower P3 amplitudes (Tascilar et al., 2011), it is important to consider reducing the degree of obesity suffered by individuals in order to help them achieve higher levels of cognitive functioning. Moreover, our findings of different P3 amplitudes for the incongruent and neutral conditions in the normal-weight groups but not the obese groups are similar to those of a study conducted by Kamijo et al. (2012b), who reported that only healthy weight individuals, but not their obese counterparts, exhibited greater P3 topographic distributions for tasks involving or not involving inhibition (i.e., the Go/NoGo task). These findings again implied that obesity is associated with inferior inhibition. Furthermore, similar to the findings for the behavioral measures, no P3 amplitude differences were found for the NL group compared with OH and OL groups, suggesting that normal weight individuals with low fitness have lower levels of inhibition similar to those of obese populations. Taken together from an electrophysiological perspective, both reducing the level of obesity and increasing the level of cardiovascular fitness seem to be necessary for higher levels of cognitive function, regardless of the nature of cognitive functions.

Given that the majority of ERP studies related to fitness have placed an emphasis on the P3 component, our goal in this study was to further explore the N1 component. Unlike the P3 component, which is regarded as a late positive and endogenous component that represents the allocation of attentional resources, the N1 component is an early negative and exogenous component that reflects the initial extraction of visual discrimination processes (Fabiani et al., 2009). Contrary to our hypothesis based upon previous ERP studies associated with fitness (Chang et al., 2013) and obesity (Key and Dykens, 2008), we found that neither obesity nor cardiovascular fitness was associated with N1 amplitude and latency. It should be noted, however, that the Chang et al.’s study examined the level of physical activity in an older population, while the Key and Dykens (2008) study examined adults with Prader–Willi syndrome, a genetic disorder. Our results were consistent with a study by Scisco et al. (2008) that emphasized cardiovascular fitness in young adults. Accordingly, it is speculated that cardiovascular fitness and obesity may have less of an influence on the early stage of sensory information processing in healthy younger adults whose specific cognitive processing ability may have reached the ceiling level.

### Limitations and Future Directions

The virtue of the present study is that it provides an initial step in identifying the association between cardiovascular fitness and obesity in terms of their effects on inhibition by examining both behavioral and electrophysiological results; however, it should be considered in light of several limitations. First limitation is related to the current cross-sectional design of the study, which meant that only associations among cardiovascular fitness, obesity, and inhibition, rather than cause-effect relationships, could be determined. It is suggested that further research be conducted to examine the effect of fitness on inhibition among an obese population by conducting a long-term exercise program. It is also important to consider factors that may be confounded with obesity. For example, factors such as environment related to intake food and nutrition history have been shown to influence neurocognitive performance in obese populations (Duchesne et al., 2010) and therefore should be taken into consideration. Given that our scope is to examine the inhibition, we were unable to draw conclusions in regard to other aspects of executive function, such as updating, shifting, and planning. The further examination of these aspects specifically would offer a more focused understanding.

## Conclusion

The present study provides evidence regarding the associations between obesity and inhibition as well as cardiovascular fitness and inhibition. Additionally, individuals with both normal weight and high cardiovascular fitness were found to exhibit significantly better behavioral results and greater electrophysiological measurements of the later stage of cognitive function, wherein those with both obesity and low cardiovascular fitness exhibited the poorest performances in executive function. These findings may support a primary practical suggestion that in order to achieve the optimal level of cognitive function, both a normal weight and high level of cardiovascular fitness should simultaneously be maintained.

## Author Contributions

Y-KC and CZ designed the study and oversaw the data collection. T-FS, Y-KC, and CZ analyzed the data and wrote-up the initial manuscript. LC, C-HC, and F-TS assisted with analysis of the data and organize the manuscript. All authors played a part in the preparation of the manuscript at each stage of its development. All authors have read and approved the final version of the manuscript.

## Conflict of Interest Statement

The authors declare that the research was conducted in the absence of any commercial or financial relationships that could be construed as a potential conflict of interest.

## References

[B1] Alvarez J. A. Emory E. (2006). Executive function and the frontal lobes: a meta-analytic review. *Neuropsychol. Rev.* 16 17–42. 10.1007/s11065-006-9002-x 16794878

[B2] American College of Sports Medicine (2013). *ACMS’s Guidelines for Exercise Testing and Prescription*, 9th Edn. New York, NY: Lippincott Williams and Wilkins.

[B3] Anderson V. Jacobs R. Anderson P. (2008). *Executive Functions and the Frontal Lobes: A Lifespan Perspective.* Philadelphia, PA: Psychology Press.

[B4] Angevaren M. Aufdemkampe G. Verhaar H. J. Aleman A. Vanhees L. (2008). Physical activity and enhanced fitness to improve cognitive function in older people without known cognitive impairment. *Cochrane Database Syst. Rev.* 3:CD005381 10.1002/14651858.CD005381.pub3 18646126

[B5] Borg G. A. (1982). Psychophysical bases of perceived exertion. *Med. Sci. Sports Exerc.* 14 377–381. 10.1249/00005768-198205000-00012 7154893

[B6] Buck S. M. Hillman C. H. Castelli D. M. (2008). The relation of aerobic fitness to stroop task performance in preadolescent children. *Med. Sci. Sports Exerc.* 40 166–172. 10.1249/mss.0b013e318159b035 18091008

[B7] Chaddock L. Pontifex M. B. Hillman C. H. Kramer A. F. (2011). A review of the relation of aerobic fitness and physical activity to brain structure and function in children. *J. Int. Neuropsychol. Soc.* 17 975–985. 10.1017/s1355617711000567 22040896

[B8] Chang Y. K. Chu C. H. Wang C. C. Song T. F. Wei G. X. (2015a). Effect of acute exercise and cardiovascular fitness on cognitive function: an event-related cortical desynchronization study. *Psychophysiology* 52 342–351. 10.1111/psyp.12364 25308605

[B9] Chang Y. K. Chu C. H. Wang C. C. Wang Y. C. Song T. F. Tsai C. L. (2015b). Dose-response relation between exercise duration and cognition. *Med. Sci. Sports Exerc.* 47 159–165. 10.1249/MSS.0000000000000383 24870572

[B10] Chang Y. K. Huang C. J. Chen K. F. Hung T. M. (2013). Physical activity and working memory in healthy older adults: an ERP study. *Psychophysiology* 50 1174–1182. 10.1111/psyp.12089 24308044

[B11] Colcombe S. Kramer A. F. (2003). Fitness effects on the cognitive function of older adults: a meta-analytic study. *Psychol. Sci.* 14 125–130. 10.1111/1467-9280.t01-1-01430 12661673

[B12] Dai C. T. Chang Y. K. Huang C. J. Hung T. M. (2013). Exercise mode and executive function in older adults: an ERP study of task-switching. *Brain Cogn.* 83 153–162. 10.1016/j.bandc.2013.07.007 23994460

[B13] Duchesne M. Mattos P. Appolinario J. C. de Freitas S. R. Coutinho G. Santos C. (2010). Assessment of executive functions in obese individuals with binge eating disorder. *Rev. Bras. Psiquiatr.* 32 381–388. 10.1590/S1516-44462010000400011 21308259

[B14] Dupuy O. Gauthier C. J. Fraser S. A. Desjardins-Crepeau L. Desjardins M. Mekary S. (2015). Higher levels of cardiovascular fitness are associated with better executive function and prefrontal oxygenation in younger and older women. *Front. Hum. Neurosci.* 9:66 10.3389/fnhum.2015.00066 PMC4332308 25741267

[B15] Edmunds L. Waters E. Elliott E. J. (2001). Evidence based paediatrics: evidence based management of childhood obesity. *BMJ* 323 916–919. 10.1136/bmj.323.7318.916 11668139 PMC1121443

[B16] Erickson K. I. Prakash R. S. Voss M. W. Chaddock L. Hu L. Morris K. S. (2009). Aerobic fitness is associated with hippocampal volume in elderly humans. *Hippocampus* 19 1030–1039. 10.1002/hipo.20547 19123237 PMC3072565

[B17] Erickson K. I. Weinstein A. M. Sutton B. P. Prakash R. S. Voss M. W. Chaddock L. (2012). Beyond vascularization: aerobic fitness is associated with N-acetylaspartate and working memory. *Brain Behav.* 2 32–41. 10.1002/brb3.30 22574272 PMC3343297

[B18] Etnier J. L. Chang Y. K. (2009). The effect of physical activity on executive function: a brief commentary on definitions, measurement issues, and the current state of the literature. *J. Sport Exerc. Psychol.* 31 469–483. 19842543 10.1123/jsep.31.4.469

[B19] Fabiani M. Gratton G. Federmeier K. D. (2009). “Event-related brain potentials: methods, theory, and applications,” in *Handbook of Psychophysiology*, eds Cacioppo J. T. Tassinary L. G. Berntson G. G. (New York, NY: Cambridge University Press), 85–119.

[B20] Fitzpatrick S. Gilbert S. Serpell L. (2013). Systematic review: are overweight and obese individuals impaired on behavioural tasks of executive functioning? *Neuropsychol. Rev.* 23 138–156. 10.1007/s11065-013-9224-7 23381140

[B21] Fletcher G. F. Balady G. J. Amsterdam E. A. Chaitman B. Eckel R. Fleg J. (2001). Exercise standards for testing and training: a statement for healthcare professionals from the American Heart Association. *Circulation* 104 1694–1740. 10.1161/hc3901.095960 11581152

[B22] Fong D. Y. Chi L. K. Li F. Chang Y. K. (2014). The benefits of endurance exercise and Tai Chi Chuan for the task-switching aspect of executive function in older adults: an ERP Study. *Front. Aging Neurosci.* 6:295 10.3389/fnagi.2014.00295 PMC4211410 25389403

[B23] Friedman D. Nessler D. Cycowicz Y. M. Horton C. (2009). Development of and change in cognitive control: a comparison of children, young adults, and older adults. *Cogn. Affect. Behav. Neurosci.* 9 91–102. 10.3758/CABN.9.1.91 19246330 PMC2692196

[B24] Golding J. M. Siegel J. M. Sorenson S. B. Burnam M. A. Stein J. A. (1989). Social support sources following sexual assault. *J. Community Psychol.* 17 92–107. 10.1002/1520-6629(198901)17:1<92::AID-JCOP2290170110>3.0.CO;2-E

[B25] Gunstad J. Lhotsky A. Wendell C. R. Ferrucci L. Zonderman A. B. (2010). Longitudinal examination of obesity and cognitive function: results from the Baltimore longitudinal study of aging. *Neuroepidemiology* 34 222–229. 10.1159/000297742 20299802 PMC2883839

[B26] Heinemann A. Kunde W. Kiesel A. (2009). Context-specific prime-congruency effects: on the role of conscious stimulus representations for cognitive control. *Conscious. Cogn.* 18 966–976. 10.1016/j.concog.2009.08.009 19796967

[B27] Hillman C. H. Belopolsky A. V. Snook E. M. Kramer A. F. McAuley E. (2004). Physical activity and executive control: implications for increased cognitive health during older adulthood. *Res. Q. Exerc. Sport* 75 176–185. 10.1080/02701367.2004.10609149 15209336

[B28] Hillman C. H. Castelli D. M. Buck S. M. (2005). Aerobic fitness and neurocognitive function in healthy preadolescent children. *Med. Sci. Sports Exerc.* 37 1967–1974. 10.1249/01.mss.0000176680.79702.ce 16286868

[B29] Hillman C. H. Kramer A. F. Belopolsky A. V. Smith D. P. (2006). A cross-sectional examination of age and physical activity on performance and event-related brain potentials in a task switching paradigm. *Int. J. Psychophysiol.* 59 30–39. 10.1016/j.ijpsycho.2005.04.009 16413382

[B30] Hsu C. L. Voss M. W. Best J. R. Handy T. C. Madden K. Bolandzadeh N. (2015). Elevated body mass index and maintenance of cognitive function in late life: exploring underlying neural mechanisms. *Front. Aging Neurosci.* 7:155 10.3389/fnagi.2015.00155 PMC4539697 26347646

[B31] Kamijo K. Khan N. A. Pontifex M. B. Scudder M. R. Drollette E. S. Raine L. B. (2012a). The relation of adiposity to cognitive control and scholastic achievement in preadolescent children. *Obesity (Silver Spring)* 20 2406–2411. 10.1038/oby.2012.112 22546743 PMC3414677

[B32] Kamijo K. Pontifex M. B. Khan N. A. Raine L. B. Scudder M. R. Drollette E. S. (2012b). The association of childhood obesity to neuroelectric indices of inhibition. *Psychophysiology* 49 1361–1371. 10.1111/j.1469-8986.2012.01459.x 22913478

[B33] Key A. P. Dykens E. M. (2008). ‘Hungry Eyes’: visual processing of food images in adults with Prader-Willi syndrome. *J. Intellect. Disabil. Res.* 52(Pt 6), 536–546. 10.1111/j.1365-2788.2008.01062.x 18422527

[B34] Liang J. Matheson B. E. Kaye W. H. Boutelle K. N. (2014). Neurocognitive correlates of obesity and obesity-related behaviors in children and adolescents. *Int. J. Obes.* 38 494–506. 10.1038/ijo.2013.142 PMC4456183 23913029

[B35] Martin A. Saunders D. H. Shenkin S. D. Sproule J. (2014). Lifestyle intervention for improving school achievement in overweight or obese children and adolescents. *Cochrane Database Syst. Rev.* 3:CD009728 10.1002/14651858.CD009728.pub2 24627300

[B36] Miller A. L. Lee H. J. Lumeng J. C. (2015). Obesity-associated biomarkers and executive function in children. *Pediatr. Res.* 77 143–147. 10.1038/pr.2014.158 25310758 PMC4416088

[B37] Miyake A. Friedman N. P. Emerson M. J. Witzki A. H. Howerter A. Wager T. D. (2000). The unity and diversity of executive functions and their contributions to complex “frontal lobe” tasks: a latent variable analysis. *Cogn. Psychol.* 41 49–100. 10.1006/cogp.1999.0734 10945922

[B38] Nederkoorn C. Coelho J. S. Guerrieri R. Houben K. Jansen A. (2012). Specificity of the failure to inhibit responses in overweight children. *Appetite* 59 409–413. 10.1016/j.appet.2012.05.028 22664299

[B39] Ng M. Fleming T. Robinson M. Thomson B. Graetz N. Margono C. (2014). Global, regional and national prevalence of overweight and obesity in children and adults 1980-2013: a systematic analysis for the Global Burden of Disease Study 2013. *Lancet* 384 766–781. 10.1016/S0140-6736(14)60460-8 24880830 PMC4624264

[B40] Nilsson L. G. Nilsson E. (2009). Overweight and cognition. *Scand. J. Psychol.* 50 660–667. 10.1111/j.1467-9450.2009.00777.x 19930267

[B41] Polich J. (2007). Updating P300: an integrative theory of P3a and P3b. *Clin. Neurophysiol.* 118 2128–2148. 10.1016/j.clinph.2007.04.019 17573239 PMC2715154

[B42] Pontifex M. B. Raine L. B. Johnson C. R. Chaddock L. Voss M. W. Cohen N. J. (2011). Cardiorespiratory fitness and the flexible modulation of cognitive control in preadolescent children. *J. Cogn. Neurosci.* 23 1332–1345. 10.1162/jocn.2010.21528 20521857

[B43] Prickett C. Brennan L. Stolwyk R. (2014). Examining the relationship between obesity and cognitive function: a systematic literature review. *Obes. Res. Clin. Pract.* 9 93–113. 10.1016/j.orcp.2014.05.001 25890426

[B44] Reinert K. R. Po’e E. K. Barkin S. L. (2013). The relationship between executive function and obesity in children and adolescents: a systematic literature review. *J. Obes.* 2013:820956 10.1155/2013/820956 PMC3595670 23533726

[B45] Scherder E. Scherder R. Verburgh L. Königs M. Blom M. Kramer A. F. (2014). Executive functions of sedentary elderly may benefit from walking: a systematic review and meta-analysis. *Am. J. Geriatr. Psychiatry* 22 782–791. 10.1016/j.jagp.2012.12.026 23636004

[B46] Scisco J. L. Leynes P. A. Kang J. (2008). Cardiovascular fitness and executive control during task-switching: an ERP study. *Int. J. Psychophysiol.* 69 52–60. 10.1016/j.ijpsycho.2008.02.009 18417237

[B47] Sibley B. A. Etnier J. L. (2003). The relationship between physical activity and cognition in children: a meta-analysis. *Pediatr. Exerc. Sci.* 15 243–256.

[B48] Siervo M. Arnold R. Wells J. C. Tagliabue A. Colantuoni A. Albanese E. (2011). Intentional weight loss in overweight and obese individuals and cognitive function: a systematic review and meta-analysis. *Obes. Rev.* 12 968–983. 10.1111/j.1467-789X.2011.00903.x 21762426

[B49] Smiley-Oyen A. L. Lowry K. A. Francois S. J. Kohut M. L. Ekkekakis P. (2008). Exercise, fitness, and neurocognitive function in older adults: the “selective improvement” and “cardiovascular fitness” hypotheses. *Ann. Behav. Med.* 36 280–291. 10.1007/s12160-008-9064-5 18825471 PMC2748860

[B50] Smith P. J. Blumenthal J. A. Hoffman B. M. Cooper H. Strauman T. A. Welsh-Bohmer K. (2010). Aerobic exercise and neurocognitive performance: a meta-analytic review of randomized controlled trials. *Psychosom. Med.* 72 239–252. 10.1097/PSY.0b013e3181d14633 20223924 PMC2897704

[B51] Stanek K. M. Strain G. Devlin M. Cohen R. Paul R. Crosby R. D. (2013). Body mass index and neurocognitive functioning across the adult lifespan. *Neuropsychology* 27 141–151. 10.1037/a0031988 23527642 PMC8674809

[B52] Stroop J. R. (1935). Studies of interference in serial verbal reactions. *J. Exp. Psychol.* 18 643–662. 10.1037/h0054651

[B53] Tascilar M. E. Turkkahraman D. Oz O. Yucel M. Taskesen M. Eker I. (2011). P300 auditory event-related potentials in children with obesity: is childhood obesity related to impairment in cognitive functions? *Pediatr. Diabetes* 12 589–595. 10.1111/j.1399-5448.2010.00748.x 21418454

[B54] Themanson J. R. Hillman C. H. (2006). Cardiorespiratory fitness and acute aerobic exercise effects on neuroelectric and behavioral measures of action monitoring. *Neuroscience* 141 757–767. 10.1016/j.neuroscience.2006.04.004 16713115

[B55] Vaughan S. Wallis M. Polit D. Steele M. Shum D. Morris N. (2014). The effects of multimodal exercise on cognitive and physical functioning and brain-derived neurotrophic factor in older women: a randomised controlled trial. *Age Ageing* 43 623–629. 10.1093/ageing/afu010 24554791

[B56] Voss M. W. Heo S. Prakash R. S. Erickson K. I. Alves H. Chaddock L. (2013). The influence of aerobic fitness on cerebral white matter integrity and cognitive function in older adults: results of a one-year exercise intervention. *Hum. Brain Mapp.* 34 2972–2985. 10.1002/hbm.22119 22674729 PMC4096122

[B57] Wechsler D. (1997). *WAIS-III, Wechsler Adult Intelligence Scale: Administration and Scoring Manual.* San Antonio, TX: Psychological Corporation.

[B58] Weinstein A. M. Voss M. W. Prakash R. S. Chaddock L. Szabo A. White S. M. (2012). The association between aerobic fitness and executive function is mediated by prefrontal cortex volume. *Brain Behav. Immun.* 26 811–819. 10.1016/j.bbi.2011.11.008 22172477 PMC3321393

[B59] Wirt T. Hundsdorfer V. Schreiber A. Kesztyus D. Steinacker J. M. (2014). Associations between inhibitory control and body weight in German primary school children. *Eat. Behav.* 15 9–12. 10.1016/j.eatbeh.2013.10.015 24411742

[B60] Wostmann N. M. Aichert D. S. Costa A. Rubia K. Moller H. J. Ettinger U. (2013). Reliability and plasticity of response inhibition and interference control. *Brain Cogn.* 81 82–94. 10.1016/j.bandc.2012.09.010 23174432

